# Antagonistic Toxic Effects of Surfactants Mixtures to Bacteria *Pseudomonas putida* and Marine Microalgae *Phaeodactylum tricornutum*

**DOI:** 10.3390/toxics11040344

**Published:** 2023-04-05

**Authors:** Francisco Ríos, Manuela Lechuga, Ismael Lobato-Guarnido, Mercedes Fernández-Serrano

**Affiliations:** Department of Chemical Engineering, Faculty of Sciences, University of Granada, Campus Fuente Nueva s/n, 18071 Granada, Spain

**Keywords:** toxicity, amine-oxide-based surfactants, ether carboxylic derivative surfactants, mixtures, *Pseudomonas putida*, *Phaeodactylum tricornutum*, antagonism, model of toxic units

## Abstract

Surfactants can be found in an ever-widening variety of products and applications, in which the combination of several types of surfactants is used to reinforce their properties, looking for synergistic effects between them. After use, they tend to be discarded into wastewater, ending up in aquatic bodies with concerning harmful and toxic effects. The aim of this study is the toxicological assessment of three anionic surfactants (ether carboxylic derivative, EC) and three amphoteric surfactants (amine-oxide-based, AO), individually and in binary mixtures of them (1:1 *w*/*w*), to bacteria *Pseudomonas putida* and marine microalgae *Phaeodactylum tricornutum*. Critical Micelle Concentration (CMC) was determined to demonstrate the capacity to reduce surface tension and the toxicity of the surfactants and mixtures. Zeta potential (ζ-potential) and micelle diameter (MD) were also determined to confirm the formation of mixed surfactant micelles. The Model of Toxic Units (MTUs) was used to quantify the interactions of surfactants in binary mixtures and to predict if the concentration addition or response addition principle can be assumed for each mixture. The results showed a higher sensitivity of microalgae *P. tricornutum* to the surfactants tested and their mixtures than bacteria *P. putida*. Antagonism toxic effects have been detected in the mixture of EC + AO and in one binary mixture of different AOs; this is to say, the mixtures showed lower toxicity than expected.

## 1. Introduction

Surfactants are commonly used in a wide range of products, including soaps, detergents, shampoos, and cleaning agents, or in agricultural, industrial, or other commercial applications. The global demand for surfactants has increased by around 300%, exceeding the current world production of 3 million tons. per year [1]. After use, mainly in aqueous solutions, they are discharged into water streams, reaching wastewater treatment plants and aquatic bodies [2]. Due to their surface-active properties, and depending on their concentration, they are toxic to aquatic living organisms causing membrane disruption, depolarization of cellular membranes or interaction with vital enzymatic proteins [3,4,5,6]. This makes the evaluation of the toxic effects of surfactants mandatory to ensure that they are safely and responsibly used. Ecotoxicity studies are necessary to understand the risks associated with the use of these compounds and to develop safer and more sustainable alternatives to traditional surfactants.

Mixtures of different surfactants (anionic, non-ionic and amphoteric surfactants) are commonly used in formulations because of their better performance compared with individual surfactants. Suitable mixtures of surfactants are mostly used to increase efficiency and stability or to reduce costs and the potential of the products to cause skin and eye irritation [7,8]. Consequently, after their use, mixtures of surfactants of a different nature are discharged together in water streams. 

In recent years, several toxicological studies concerning pollutants have focused on the joint toxicological assessment of co-pollutants, revealing that multiple interactions between toxics can appear and need to be described [9,10,11,12,13]. In the case of surfactants, while individual studies of them can provide valuable information, they may not reflect real-world exposure to multiple surfactants at once. As happens with their physicochemical properties, synergistic or antagonistic effects can appear on the combined toxicity of surfactants, meaning that the combined effects are greater or lower than the sum of the individual effects [14,15,16,17]. The toxicity of individual surfactants has been extensively studied on several aquatic organisms, such as bacteria, *Vibrio fisheri*, microalgae, *Pseudokirchneriella subcapitata*, or microcrustaceans, *Daphnia magna*, especially for the traditional and more common surfactants [2,18,19]. Nevertheless, there is a lack of research that studies the combined effects of mixtures of surfactants and evaluates the possibility of synergistic, additive, or antagonistic effects. In the case of mixtures that exhibit synergistic effects, surfactants may interact with each other, and the combined toxicity of the surfactants is greater than the sum of their individual toxicities. If there is an additive effect, it means that the combined toxicity is equal to the sum of their individual toxicities. On the other hand, in case of an antagonistic effect, surfactants may interact being the mixture less toxic than the sum of their individual toxicities, which is very relevant from an eco-friendly point of view [20].

To address this gap in knowledge, it is important to conduct research that examines the effects of multiple surfactants and focuses on the elucidation of the type of action of these mixtures on aquatic organisms. The model of toxic units (MTU) [20,21,22] is used to quantify the interactions of toxicants in binary mixtures of chemicals and to predict if the concentration/dose addition principles can be assumed, meaning that both surfactants act by the equal mode of action (MoA). Equally, it allows us to predict if the response addition principle can be expected; this is, surfactants act independently from each other, usually by different MoAs that do not influence each other [15].

Among commercial surfactants, ether carboxylic derivative surfactants are frequently used in cleaning, personal care, and cosmetic formulations, providing an excellent foaming capacity and low eye and skin irritation potential [23]. Amine-oxide-based surfactants are excellent foaming stabilizers in mixtures of anionic and amphoteric surfactants. Its addition, they increase the detergency level of the mixture, being commonly used in cosmetic formulations, washing up liquids, and cleaners in general. The individual toxicity of both families of surfactants to bacteria *V. fischeri*, freshwater microalgae, and microcrustaceans have been previously reported [16,18,23]. Nevertheless, toxicity to other relevant and representative species, such as *Pseudomonas* or marine microalgae, has not been studied, and more importantly, the mode of action and possible additive, synergistic or antagonistic effects of these surfactant mixtures has not ever been addressed. 

In this study, we evaluated the toxicity of three anionic surfactants (ether carboxylic derivative, EC) and three amphoteric surfactants (amine-oxide-based, AO), individually and in binary mixtures (1:1 *w*/*w*), to bacteria *Pseudomonas putida* and marine microalgae *Phaeodactylum tricornutum*. *P. putida*, which are Gram-negative bacteria that can be found in surface waters and are commonly used in environmental studies because of their capacity to degrade a wide range of organic compounds and their resistance to environmental stressors [24,25]. They are frequently used for toxicity assessments, and a standard toxicity procedure has been proposed for the evaluation of the growth inhibition of these bacteria caused by toxicants [26,27]. They can also be considered to be representative heterotrophic microorganisms in freshwater and as a simplified model of an activated sludge biotreatment. In addition, the use of *P. putida* is preferable to some other Pseudomonas species as it is a safe species of bacteria, unlike *P. aeruginosa*, for example, which is an opportunistic human pathogen. Marine microalgae *Phaeodactylum tricornutum* are important primary producers in marine ecosystems and are sensitive indicators of environmental stressors, including toxic substances. Toxicity testing with marine microalgae *P. tricornutum* is a common method used to assess the potential impact of substances on marine aquatic environments in a rapid, sensitive, and cost-effective way [28,29,30,31,32]. 

This study extends the knowledge concerning the toxicity of individual anionic surfactants (EC) and amphoteric surfactants (AO) and binary mixtures of them (anionic/anionic, anionic/amphoteric and amphoteric/amphoteric) with two representative organisms from fresh and marine water. Additionally, especially, it contributes to increasing the knowledge concerning the type of action of the surfactants and identifies potential interactions between surfactants, providing more accurate information concerning the risks associated with exposure of aquatic organisms to mixtures of surfactants. This brings valuable information for the selection of surfactants in the formulation of more eco-friendly products. 

## 2. Materials and Methods

### 2.1. Surfactants

**Figure 1:**
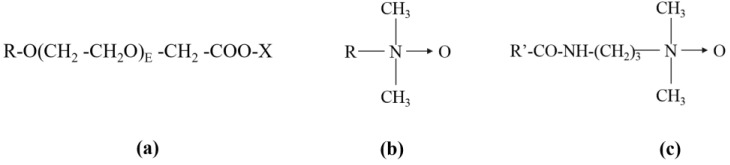
Molecular structure (**a**) ether carboxylic derivative surfactants, EC-R_8_E_8_, EC-R_12–14_E_3_, EC-R_12–14_E_10_, (**b**) amine-oxide-based surfactants, AO-R_14_ and AO-R_12_, (**c**) amine-oxide-based surfactant, AO-_Cocoamido_. R: length alkyl chain; E: ethoxylation degree, R’:R_12_.

The toxicity of three anionic surfactants (ether carboxylic derivative surfactants) and three amphoteric surfactants (amine-oxide-based surfactants) supplied by Kao Chemicals Europe (Barcelona, Spain) was tested. Amine-oxide-based surfactants are a special class of surfactants that have cationic behavior under acid conditions and a non-ionic character in neutral and basic mediums (pH range of the toxicity tests later described). Figure 1 shows their molecular structure, and Table 1 shows their abbreviation name, INCI name, CAS registry number, length alkyl chain (R), ethoxylation degree (E), and % active matter. 

### 2.2. Reagents

The reagents used in the toxicity tests were of analytical grade, supplied by MercK KGaA (Darmstadt, Germany). The solutions were prepared in ultrapure MilliQ^®^ water (resistivity 18.2 MΩ·cm at 25 °C). The glassware in contact with surfactants was carefully cleaned with a dissolution of 16 gL^−1^ of ammonium peroxydisulfate in sulfuric acid (98%).

### 2.3. Active Matter Analysis

The active matter of the surfactants tested was measured by infrared radiation (model AD-4714A, A&D, Tokyo, Japan). The temperature of drying was set at 105 °C. The water content in the sample was registered every 30 s for 90 min as the difference from the initial weight. Active matter is calculated as 100% minus the water content determined (Table 1).

### 2.4. Critical Micelle Concentration

The surfactant concentration at which surfactant molecules can aggregate and form micelles is known as the critical micelle concentration (CMC). The surface tension of a concentration series (ranging from 0.1 to 1 × 10^4^ mgL^−1^) was measured in order to calculate the CMC. Surface tension plots, as a function of surfactant concentration in a semi-log plot, result in a quick linear decline in surface tension, followed by a gradual decline. The production of micelles (CMC) is indicated by the breakpoint. The Wilhelmy Plate Method was used to measure surface tension using a tensiometer model K11 (Krüss GmbH, Hamburg, Germany) fitted with a 2 cm platinum plate, in accordance with the BS EN 14370:2004 guidelines [33]. Before each measurement, the plate was carefully cleaned and flame-dried with a Bunsen burner. By sinking a vertical plate into the solution and then pulling it out again, the surface tension was measured. The standard deviation did not surpass 0.1 mNm^−1^ during the six subsequent observations. The jacketed cell was filled with thermostated water, which maintained the temperature at 25 ± 0.5 °C. CMC determinations were performed in triplicate to obtain a mean CMC and its confidence interval (95%).

### 2.5. Zeta Potential (ζ-Potential) and Micelle Diameter (MD)

The Zeta potential (a measure of the magnitude of the electrostatic or charge repulsion/attraction between micelles) of the individual and mixtures of surfactants (1:1 *w*/*w*) under the conditions of the toxicity test of *P. putida* (freshwater) and *P. tricornutum* (marine medium) was analyzed using a Zetasizer Ultra (Malvern Panalytical Ltd., Malvern, UK) by the technique of Electrophoretic Light Scattering (ELS). The Zetasizer system was also used to measure the average micellar hydrodynamic diameter (MD) of the surfactants tested above the CMC in the two-culture media by dynamic light scattering (DLS). The detection limit of the equipment is 0.3 nm to 10 mm. The instrument settings were optimized automatically by means of the ZS XPLORER software (Malvern Panalytical Ltd., Malvern, UK). All of the experiments were performed at a controlled temperature (25 ± 1 °C) in triplicate. 

### 2.6. Pseudomonas Putida Toxicity Test

The *P. putida* toxicity test was carried out following the guideline ISO 10712:1995 (Water quality—*Pseudomonas putida* growth inhibition test [26]. A monoculture strain *Pseudomonas putida* CECT 324, provided by the Spanish Type Culture Collection (València, Spain), was used in the toxicity tests. Bacteria were supplied in a lyophilized form. These cultures were revived on a solid agar Petri dish at pH 7.2, 1 gL^−1^ beef extract, 2 gL^−1^ yeast extract, 5 gL^−1^ peptone, 5 gL^−1^ NaCl, and 15 gL^−1^ agar powder.

*P. putida* was incubated on the surface of the solid stock culture (1 gL^−1^ NaNO_3_, 0.24 gL^−1^ K_2_HPO_4_, 0.12 gL^−1^ KH_2_PO_4_, 0.1 gL^−1^ yeast extract, 10 gL^−1^ glucose, 0.4 gL^−1^ MgS0_4_·7H_2_O, 0.001 gL^−1^ ferric citrate and 18 gL^−1^ agar powder) in slant tubes for 24 h. After the incubation period, the cells were rinsed with a preculture medium (0.5 gL^−1^ NaNO_3_, 0.12 gL^−1^ K_2_HPO_4_, 0.06 gL^−1^ KH_2_PO_4_, 0.05 gL^−1^ yeast extract, 2 gL^−1^ glucose, 0.2 gL^−1^ MgS04·7H_2_O and 0.0005 gL^−1^ ferric citrate) and transferred into a flask with a turbidity of 10 FNU (A610 nm, 0.02) using preculture medium. This inoculum was incubated for 7 days at 25 ± 1 °C with constant agitation. The inoculum was added to the preculture medium and incubated for 5 ± 0.5 h at 23 ± 1 °C in a shaker. When the incubation ended, the bacterial suspension was diluted to 50 FNU (A610 nm, 0.1). Furthermore, 10 mL of this preculture was used to inoculate the flasks with different surfactant concentrations. The pH of the solutions was adjusted to 7.4 ± 0.3 with either 1 N HCl or 1 M NaOH before the assay was initiated.

The flasks were incubated in the dark for 16 h at 23 ± 1 °C in a shaker. After the incubation, the turbidity in the test flask was measured using a spectrophotometer at 610 nm. 

The growth inhibition was evaluated as the change in the absorbance of the bacterial culture before and after exposure. The percentage of the inhibition of cell multiplication (*I*) for each concentration of the tested surfactant was calculated using the following equation:

(1) $$I\left(\%\right)=\frac{B_{C}-B_{n}}{B_{C}-B_{0}}\cdot 100$$

where *I* is the cell multiplication inhibition [%], *B_n_* is the turbidity of the biomass measured after the test time for the umpteenth concentration in the test flask, *B_c_* is the turbidity of the biomass measured after the test time in the control flask, and *B*_0_ is the value of the turbidity of the biomass measured at time 0 in the control flask. The values of *I* were plotted against the surfactant concentrations. The toxicity values were measured as *EC*_50_, which is the surfactant concentration that inhibits 50% after 16 h of exposure. 

### 2.7. Marine Algae Toxicity Test

The 72 h algal growth-inhibition test with the marine algal *Phaeodactylum tricornutum* was administered according to the Guideline ISO 10253:2006 (Water quality—Marine algal growth inhibition test with *Skeletonema costatum* and *Phaeodactylum tricornutum*) [34]. The microalgae inoculum, supplied by Microbiotest Inc. (Gent, Belgium), was incubated for 3 days at 20 ± 2 °C, with constant uniform illumination (10,000 lux) in a growth medium prepared with the addition of 1 L: 22 g NaCl, 9.7 g MgCl_2_·6H_2_O, 3.7 g Na_2_SO_4_, 1.0 g CaCl_2_, 0.65 g KCl, 0.2 g NaHCO_3_ and 40.1 mg H_3_BO_3_, 0.72 mg FeCI_3_·6H_2_O, 2.16 mg MnCI_2_·4H_2_O, 0.68 mg ZnSO_4_·7H_2_O, 2.4 µg CuSO_4_·5H_2_O, 6.1 µg CoCI_2_·6H_2_O, 15 mg Na_2_EDTA, 25 µg Thiamin hydrochloride, 0.05 µg biotin, 0.05 µg vitamin B_12_, 3 µg K_3_PO_4_, 40 µg NaNO_3_, and 14.9 µg Na 2SiO_3_·5H_2_O. After 3 days of incubation, the inoculum of the algae was added to the vials with the substance to be tested and to the control vials to obtain an algae concentration of 1·10^4^ algae cells mL^−1^. Five concentrations of the target substance and control were performed in triplicate in 10 cm path length vials. The vials were kept at 20 ± 2 °C under constant uniform illumination (10,000 lux) for 72 h, and the pH was set to 8.0 ± 0.2 with either 1 N HCl or 1 M NaOH.

After 24, 48, and 72 h, the optical density of the culture at 670 nm was determined to establish whether growth had been inhibited or stimulated with respect to the control. Specific growth rates (*µ*) were calculated using Equation (2).

(2) $$\mu_{i}\left(h^{-1}\right)=\frac{lnN_{L}-lnN_{0}}{t_{L}-t_{0}}$$

where *t*_0_ is the time of the test start, *t_L_* is the time of the test determination (24, 48, and 72 h), *N*_0_ is the nominal initial cell density, and *N_L_* is the measured cell density at time *t_L_*.

The percentage inhibition for each test concentration (*I_µi_*) was calculated from Equation (3):

(3) $$I_{\mu i}\left(\%\right)=\frac{\mu_{c}-\mu_{i}}{\mu_{c}}\cdot 100$$

where *µ_c_* is the mean growth rate for the control and *µ_i_* the growth rate for test vial *i*. 

A linear relationship can be deduced between the inhibition and the concentration of the surfactant used in the following form:

(4) $$\log\left(i\right)=A\cdot I+B$$

*EC*_50_ represents the concentration of surfactant that caused a 50% growth inhibition after 72 h of exposure. Three replicate tests were performed to obtain a mean *EC*_50_ and its confidence interval (95%).

### 2.8. Model of Toxic Units

The model of toxic units (MTU) [20,21,22] was used to quantify the interactions of the toxicants and to predict if the concentration/dose or response addition principles can be assumed. *TU_i_* is the relation of the concentration of the toxicant in a mixture [*i*] and its half maximal effective concentration (*EC*_50*i*_), (Equation (5)) being the toxic unit of the mixture (*TU_mix_*), the sum of *TU_i_* of the single components (Equation (6)).

(5) $${TU}_{i}=\frac{\left[i\right]}{{EC}_{50i}}$$

(6) $${TU}_{mix}={TU}_{A}+{TU}_{B}=\frac{\left[A\right]}{{EC}_{50A}}+\frac{\left[B\right]}{{EC}_{50B}}$$

According to the bibliography and previous studies, simple additivity (concentration addition) is characterized by 0.8 < *TU_mix_* < 1.2, while *TU_mix_* ≤ 0.8 indicates synergism (more than additive) and *TU_mix_* ≥ 1.2 represents antagonism (less than additive) [35,36,37].

If toxicants work independently from each other (response addition), the predicted toxic unit (*TU_r_*) needs to be calculated by Equations (7) and (8). When *TU_r_* ≈ 1, the response addition can be expected [33].

(7) $$\mathrm{If}\;{\mathrm{TU}}_{A}>\;{\mathrm{TU}}_{B}\;{\mathrm{TU}}_{r}=1+\frac{{TU}_{B}}{{TU}_{A}}$$

(8) $$\mathrm{If}\;<\;{{\mathrm{TU}}_{B}}_{\;}{\mathrm{TU}}_{r}=1+\frac{{TU}_{A}}{{TU}_{B}}$$

### 2.9. Statistical Analysis

Statistical analysis was performed using the statistical program Statgraphics 5.1. The data were subjected to analysis of variance (ANOVA). Tukey’s multiple range test [38] was used to determine if significant differences existed between the groups of toxicity data to bacteria *P. putida* and microalgae *P. tricornutum*. The differences between the mean values were considered significant at a level of confidence of 95%.

## 3. Results

### 3.1. Critical Micelle Concentration (CMC)

CMC is a very relevant parameter in the case of toxicity studies of surfactants, as it indicates if the surface tension in the solution has been reduced to the minimum, concentration above (CMC), or surfactant monomers are only situated in the air–water interface (below CMC), which can affect the availability of surfactant molecules to partition into cellular membranes and cause toxicity. Higher toxicity is correlated with higher hydrophobicity [39] but is also dependent on CMC since toxic effects, specifically for non-ionic surfactants, take place at surfactant concentrations near or above the CMC [40,41]. The CMC values of the surfactants studied have been reported in previous studies [16,23]; nevertheless, CMCs have been again determined with the batch of surfactants tested in this study. CMCs for the mixtures (1:1 *w*/*w*) have also been determined, and the parameter of the CMCU (Critical Micelle Concentration Unit) has been calculated as the relationship between the CMC obtained for the mixture and the average CMC values for the individual surfactants. Table 2 shows the CMC and CMCU values determined for the individual surfactants and the mixtures tested. 

### 3.2. Zeta Potential and Micelle Diameter

The ζ-potential and average MD of the individual and surfactant mixtures (1:1 *w*/*w*) in the growth medium of *P. putida* and *P. tricornutum* have been measured to confirm the formation of mixed micelles and to understand the effect of the growth medium on the stability and size of the micelle formed. The results are shown in Table 3, including the standard deviation (SD) of three replicates.

### 3.3. Toxicity of Individual Surfactants

The toxicity of the individual anionic and the amphoteric surfactants to bacteria *P. putida* (exposure time: 16 h) and marine microalgae *P. tricornutum* (exposure time: 72 h) was determined. The results are shown in Table 4. The toxicity values are indicated as *EC*_50_ (concentration of the substance tested in water causing a 50% of growth inhibition). The values ranged from 28.8 mgL^−1^ to 443.95 mgL^−1^ for *P. putida* and from 4.27 mgL^−1^ to 93.05 mgL^−1^ in the case of *P. tricornutum*. The results reveal a higher sensitivity of the marine microalgae *P. tricornutum* than bacteria *P. putida* for the surfactants tested.

### 3.4. Toxicity of Surfactants Mixtures

Table 5 and Table 6 show the toxicity values of the mixtures (1:1 *w*/*w*) of the surfactants tested to bacteria *P. putida* (exposure time: 16 h) and marine microalgae *P. tricornutum* (exposure time: 72 h), respectively. They include a mixture of two anionic surfactants (EC-R_8_E_8_ + EC-R_12–14_E_10_), a mixture of an ether carboxylic derivative surfactant and the less toxic amine-oxide-based surfactants (EC-R_8_E_8_ + AO-_Cocoamido_), and two binary mixtures of the amphoteric surfactants (AO-R_12_ + AO-_Cocoamido_ and AO-R_14_ + AO-_Cocoamido_). The toxicity of mixtures using the anionic surfactant EC-R_12–14_E_3_ is not included since the solutions of this surfactant with the other ether carboxylic derivative surfactants and amine-oxide-based surfactants showed turbidity in the concentration range of the tests, making it impossible to determine the growth inhibition of bacteria or microalgae by spectrophotometric measurements. In the same way, it was not possible to determine the toxicity of the mixture of AO-R_12_ and AO-R_14_. 

Using the model of Toxics Units previously described [20,21,22], the toxic units of the single components (*TU_A_, TU_B_*), toxic units of the mixtures (*TU_mix_*), and the predicted toxic units (*TU_r_*) were calculated. The values, together with the conclusions on the type of action for the mixtures tested to bacteria *P. putida* and microalgae *P. tricornutum,* are included in Table 5 and Table 6, respectively.

## 4. Discussion

### 4.1. Zeta Potential and Micelle Diameter of Individual and Mixtures of the Surfactant Solutions

All of the surfactant solutions tested showed a negative ζ-potential ranging from −51.9 to −2.4, showing the lowest value for the individual anionic surfactant EC-R_12–14_E_3_ in the *P. putida* medium (Table 3). As expected, the ζ-potential is higher for surfactants in the marine microalgae medium, indicating the lower stability of the surfactant micelles caused by the shielding effect [42,43]. Comparing the ζ-potential of the surfactant mixtures with the ζ-potential of the individual surfactants, it can be observed that approximately intermediate values were obtained for both culture media (*P. putida* and *P. tricornutum*), and this indicates the formation of mixed micelles in the mixture solutions. It can only be appreciated that for the mixture of AO-R_14_ and AO-_Cocoamido_, the ζ-potential in the *P. putida* medium is slightly above the ζ-potential of the individual surfactants, and the ζ-potential of the mixture EC-R_8_E_8_ and EC-R_12–14_E_10_ in the *P. tricornutum* medium is slightly below the ζ-potential of the individual surfactants. Nevertheless, considering the standard deviation and experimental errors, these differences can be considered to be negligible.

In the case of the MD of the individual surfactants (Table 3), the results show a higher MD for the ether carboxylic derivative surfactant with the shorter alkyl chain and lower hydrophobicity (EC-R_8_E_8_), whereas the MD of the ether carboxylic derivative with the longest alkyl chain (EC-R_12–14_E_10_) shows the lowest value in both culture media. The concentration of salts in the medium had a direct effect on the average MD for most of the surfactants; this is, the MD of the individual surfactants is higher in the *P. tricornutum* medium than in the *P. putida* medium. This could be related to the higher stability of the micelles (lower ζ-potential) in the *P. putida* medium; consequently, the possibilities of interactions between micelles to form bigger micelles are prevented. On the other hand, it is especially remarkable that the great MD of the amine-oxide-based surfactant AO-_Cocoamido_ in the *P. tricornutum* medium, which is six times higher than its MD in the *P. putida* medium, which could be influenced by the interaction of salts with the amide group existing in the fatty alkyl chain. 

Regarding the average MD for the mixtures of the surfactants, for most of them, the MD is lower than the MD of the individual surfactants or closer to the lowest value of the individual surfactants, revealing the preference of the surfactants monomers to form smaller micelles if they are included in mixed micelles. It is remarkable that in the mixture EC-R_8_E_8_ + AO-_Cocoamido_, the MD in a marine medium is much smaller than the MD of the individual surfactants. It is worth noting that, in the case of mixtures, the MD is the average of multiple possible combinations of mixed micelles containing monomers from surfactants A and B. 

### 4.2. Toxicity of Individual Surfactants

Overall, the surfactants tested are more toxic to marine microalgae *P. tricornutum* than to bacteria *P. putida* (Table 4). The values of *EC*_50_ for *P. putida* were between 3 and 40 times higher than *EC*_50_ for *P. tricornutum*. Especially relevant is the difference for the anionic surfactant EC-R_12–14_E_3_. The results contrast with some results found in similar studies with other species of the same trophic levels. In previous studies [16,35,44], we found higher toxicity of polyoxyethylene glycerol esters, ether carboxylic derivatives, alkylpolyglucosides and amine-oxide-based surfactants to marine bacteria *V. fischeri* compared with freshwater microalgae *P. subcapitata*. There are no published toxicity data available regarding the surfactants and species tested in this study for comparison. However, the differences could be related to the different behavior of the surfactants on saline and freshwater microalgae mediums, corroborated by measurements of the CMC, ζ-potential and MD in the different media (Table 2 and Table 3). In a medium with a higher concentration of salts, surfactants can easily decrease the surface tension at a lower surfactant concentration [45], which is directly related to the toxicity effect on microorganisms. In the case of microalgae, surfactants can induce morphological changes in the cells affecting the structure and integrity of lipid membranes, denaturing the membrane proteins, and consequently, increasing the permeability of the cellular membrane and causing the leakage of vital compounds [46]. In the case of bacteria, surfactants can depolarize the microbial cell membrane and decrease the absorption of nutrients and the acceptance of oxygen [6]. Nevertheless, bacteria *P. putida* also has a broad tolerance to toxic compounds and oxidative stress, and they can use surfactants as a source of carbon in their metabolic processes [25,47]. This suggests that a higher surfactant concentration is needed to exert an inhibition effect in the growth population of bacteria *P. putida* than in microalgae *P. tricornutum*.

It could be expected that the anionic surfactants show higher toxicity compared with non-ionic surfactants, as has been extensively reported in the literature for several species [19,48,49] since the ability of anionic surfactants to partition from aqueous environments into the lipid membranes of aquatic organisms is higher [50]. Nevertheless, some deviations have been found in this study. Amine-oxide-based surfactants with a non-ionic character in the toxicity media showed greater inhibition of the bacteria and microalgae growth than the ether carboxylic derivative surfactants. Similar results were found in previous studies of AO-R_12_ and AO-R_14_ to bacteria *V. fischeri* and microcrustaceans *D. magna*, where they showed higher toxicity than ether carboxylic derivative surfactants [16].

Regarding the structural parameters, ether carboxylic derivative surfactants with a longer alkyl chain(R) showed a lower value of *EC*_50_, meaning stronger toxic effects in both species; this is related to the higher hydrophobicity of the surfactants with longer alkyl chains, as indicated by their CMC (Table 2) [51]. In the case of ether carboxylic derivative surfactants with the same alkyl chain but different degrees of ethoxylation (EC-R_12–14_E_3_ and EC-R_12–14_E_10_), the surfactant with the higher degree of ethoxylation and less hydrophobic (EC-R_12–14_E_10_) presented lower toxicity. The same behavior is observed when comparing AO-R_12_ and AO-R_14_, while AO-_Cocoamido_ is the less toxic amine-oxide-based surfactant, which is directly related to the amide group existing in the fatty alkyl chain, making the surfactant molecule more hydrophilic and, therefore, less likely to cause membrane disruption [18,52]. The presence of the amine group is also reflected in its higher CMC (Table 2); the surfactant is less effective in decreasing the surface tension but is less toxic to aquatic organisms. Regarding the relationship between the structural parameters and hydrophobicity, the toxic effects can be appreciated in the same trend with both species tested, bacteria *P. putida* and marine microalgae *P. tricornutum*. 

### 4.3. Toxicity of Surfactants Mixtures

**Figure 2:**
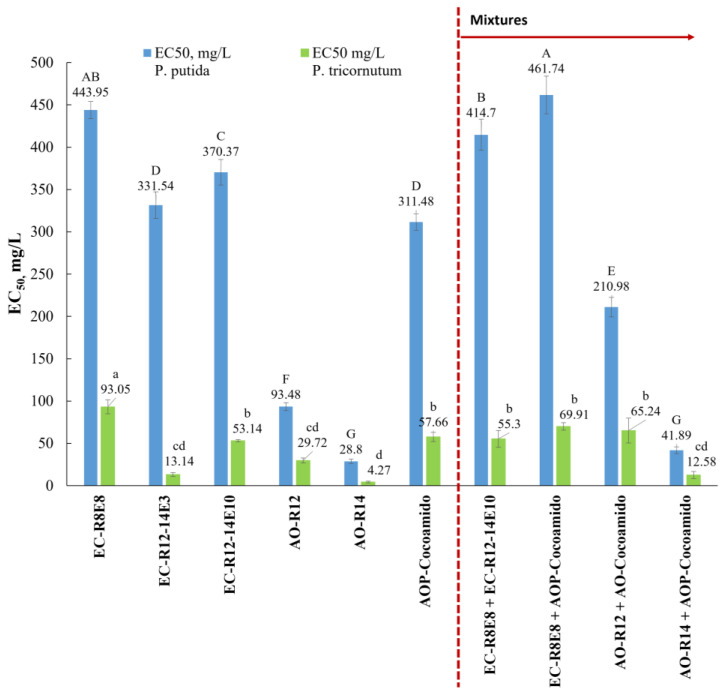
Toxicity values (95% CI) for individual and mixtures of surfactants. Mean values with different superscript letters (uppercase: *P. putida*; lowercase: *P. tricornutum*) are significantly different (*p* < 0.05).

Similar to the toxicity of individual surfactants, the surfactant mixtures had greater toxicity effects on marine microalgae *P. tricornutum* than bacteria *P. putida* (Table 5 and Table 6). The *EC*_50_ values ranged from 41.89 mgL^−1^ to 461.74 mgL^−1^ for *P. putida* and from 12.58 mgL^−1^ to 69.91 mgL^−1^ for *P. tricornutum*. The toxicity of the mixtures shows interesting results from an antagonism point of view. Figure 2 has been added to provide a clearer interpretation and a better visual appreciation of the results, including significantly different groups according to Tukey’s multiple comparison test.

Firstly, the mixture of the two anionic surfactants (EC-R_8_E_8_ + EC-R_12–14_E_10_) shows an intermediate value of *EC*_50_ compared to the *EC*_50_ of the individual surfactants for both species tested. Considering the model of toxic units and the calculated parameter *TU_mix_*, the type of action can be assumed to be the concentration addition (0.8 ≤ *TU_mix_* ≤ 1.2); this is to say, both surfactants act with the same MoA. As anionic surfactants, it is assumed that they likely act as polar narcosis class 2 [53,54,55]. The CMCU for the mixtures was 0.71 < 1; this is, the CMC of the mixture is lower than the average CMC of the individual surfactants, and therefore, the mixture is more efficient in decreasing the surface tension than the individual surfactants. Nevertheless, this effect cannot be traduced in a clear synergistic effect on the toxicity of the mixture since the calculated values of the *TU_mix_* for the mixture with both species are not below 0.8.

In the case of the mixture of EC-R_8_E_8_ + AO-_Cocoamido_, a higher value of *EC*_50_ to bacteria *P. putida* is obtained compared to the *EC*_50_ of the individual surfactants, reflecting an antagonism effect on the toxicity; this is, the mixture is less toxic than the individual surfactants. According to MTU, the type of action cannot be assumed as simple additivity (*TU_mix_* = 1.26) or response addition (*TU_r_* ≠ 1), being more likely less than additive. When testing this mixture with marine microalgae *P. tricornutum*, the concentration addition is supposed to be the type of action (*TU_mix_* = 0.98). The toxicity MoA of amine-oxide-based surfactants has been barely studied, so conclusions are difficult to draw without a deeper and more specific study [35]. The CMCU for this mixture was above 1, which means that more concentration of the surfactant mixture is needed to reach the lowest surface tension compared with the amount of the individual surfactants. 

From the toxicity results for the binary mixtures of AO-R_12_ + AO-_Cocoamido_, similar conclusions be drawn for bacteria *P. putida* and marine microalgae *P. tricornutum*, and an antagonism effect is appreciated, wherein the *EC*_50_ for the mixture is higher than the value expected. In the case of *P. putida*, a type of action that is less than additive and close to the response addition can be suggested (*TU_mix_* = 1.47, *TU_r_* = 1.30). Although both surfactants in the mixture belong to the same surfactant family, AO-R_12_ is around three times more toxic than AO-_Cocoamido_, and the toxicity effects appear to be mainly conducted by the AO-R_12_. In the case of *P. tricornutum*, a less than additive type of action could also be suggested (*TU_mix_* = 1.66), and response addition is not expected. The antagonistic effect of this mixture is also reflected in the CMC of the mixture, showing a CMCU value higher than 1; the CMC for the mixture is higher than the CMC of the individual surfactants. This means that from an environmental point of view, the mixtures of AO-R_12_ + AO-_Cocoamido_ are more eco-friendly, but from an efficacy point of view, for the decreasing surface and interfacial tension, the mixture of them is not recommended. The ζ-potential measurements of this mixture of surfactants showed an intermediate value between the ζ-potential of the individual surfactants; therefore, the theory stated by Martins et al. [56] proposing that more stable micelles lead to lower toxicity cannot be applied. (i.e., stable micelles lead to lower surfactant monomers, which are more efficient than micelles when entering the cell through cell wall pores). 

Finally, looking at the results for the binary mixture of AO-R_14_ + AO-_Cocoamido_ and *TU_r_* values, response addition can be suggested as the type of action of the mixture for both species, bacteria *P. putida* and marine microalgae *P. tricornutum* (*TU_r_* = 1.07 and 1.09, respectively). That means that each surfactant acts with a different MoA. Although the evidence is not completely clear, AO-_Cocoamido_ could be acting in a similar way to the anionic surfactant, EC-R_8_E_8_ (polar narcosis class 2) (as previously, MoA for EC-R_8_E_8_ has been suggested, and in the mixture EC-R_8_E_8_ + AO-_Cocoamido_, no response addition has been identified), whereas MoA for AO-R_14_ could be assumed to be non-polar narcosis class 1 [53]. It is worth noting that the surfactant AO-R_14_, the one with the stronger hydrophobic character, is 11 times more toxic than AO-_Cocoamido_ for bacteria *P. putida* and 13 times more toxic for marine microalgae *P. tricornutum*, so the higher contribution to the growth inhibition can be attributed to the amine-oxide-based surfactant AO-R_14_.

## 5. Conclusions

This study assessed and compared the toxic effects of ether carboxylic derivative anionic surfactants and amine-oxide-based amphoteric surfactants individually and in binary mixtures (1:1 *w*/*w*) to bacteria *P. putida* and marine microalgae *P. tricornutum*. ζ-potential and MD measurements confirmed the formation of mixed surfactant micelles and the shielding effect of the marine medium in the micelles. The toxicity results showed the higher sensitivity of microalgae *P. tricornutum* to the surfactants tested and the mixtures of them than bacteria *P. putida*, which is related to the higher capacity to decrease the surface tension of the surfactants in a marine medium [45]. The results confirmed the higher toxicity of the surfactants with higher hydrophobicity (surfactant molecules with a longer alkyl chain, a lower degree of ethoxylation, or an amide group in the fatty alkyl chain).

Considering the MTU, concentration addition has been assumed as the type of action for the binary mixture of EC-R_8_E_8_ + EC-R_12–14_E_10_ with both tested species and for the mixture of EC-R_8_E_8_ + AO-_Cocoamido_ to marine microalgae, whereas response addition could be expected as the type of action to the binary mixture of AO-R_14_ + AO-_Cocoamido_ with *P. putida* and *P. tricornutum*. Antagonism toxic effects have also been identified for some mixtures: EC-R_8_E_8_ + AO-_Cocoamido_ to bacteria *P. putida* and AO-R_12_ + AO-_Cocoamido_ to *P. putida* and *P. tricornutum.* This is to say that these mixtures showed lower toxicity than expected when looking at the *EC*_50_ values of the individual surfactants.

The most toxic mixture was AO-R_14_ + AO-_Cocoamido_, whereas mixture EC-R_8_E_8_ + AO-_Cocoamido_ was the least lethal for both species tested. The use of AO-R_12_ in formulations, and its subsequent presence in aquatic environments, is preferable rather than AO-R_14_ if an amine-oxide-based surfactant with these characteristics needs to be used since it has antagonistic toxic effects with AO-_Cocoamido_, albeit the use of the combination of EC-R_8_E_8_ + AO-_Cocoamido_ is the best option. 

## Figures and Tables

**Table 1:** Chemical identification and technical specifications of the surfactants tested.

Abbreviation	INCI Name ^1^	CAS ^2^	R ^3^	E ^4^	% Active Matter
EC-R_8_E_8_	Capryleth-9 carboxylic acid	53563-70-5	8	8	92.8
EC-R_12–14_E_3_	Laureth-4 carboxylic acid	27306-90-7	12–14	3	92.5
EC-R_12–14_E_10_	Laureth-11 carboxylic acid	27306-90-7	12–14	10	89.1
AO-R_12_	Lauryl dimethyl amine oxide	1643-20-5	12	-	29.4
AO-R_14_	Myristyl dimethyl amine oxide	3332-27-2	14	-	30.8
AOP-_Cocoamido_	Cocoamidopropyl dimethyl amine oxide	68155-09-9	R’ = 12	-	34.3

^1^ INCI: international nomenclature of cosmetics ingredients. ^2^ CAS: Chemical Abstracts Service registry number. ^3^ R: alkyl chain length, n-C_i_H_2i + 1-_. ^4^ E: degree of ethoxylation–(OCH_2_CH_2_)_n_O.

**Table 2:** CMC (95% CI) and CMCU of surfactants and surfactants mixtures (1:1 *w*/*w*).

Surfactant	CMC (mgL^−1^)	CMCU
EC-R_8_E_8_	214.2 ± 5.2	-
EC-R_12–14_E_3_	28.6 ± 4.8	-
EC-R_12–14_E_10_	66.9 ± 7.2	-
AO-R_12_	156.0 ± 10.5	-
AO-R_14_	102.4 ± 3.2	-
AOP-_Cocoamido_	301.3 ± 5.1	-
EC-R_8_E_8_ + EC-R_12–14_E_10_	100.2 ± 1.57	0.71
EC-R_8_E_8_ + AO-_Cocoamido_	442.7 ± 7.6	1.71
AO-R_12_ + AO-_Cocoamido_	403.8 ± 12.4	1.76
AO-R_14_ + AO-_Cocoamido_	157.9 ± 6.1	0.78

**Table 3:** ζ-potential and MD of surfactants and surfactants mixtures (1:1 *w*/*w*) in culture media of *P. putida* and *P. tricornutum*.

Surfactant	ζ-Potential *P. putida* Medium, (±SD)	MD *P. putida* Medium, (nm ± SD)	ζ-Potential *P. tricornutum* Medium, (±SD)	MD *P. tricornutum* Medium, (nm ± SD)
EC-R_8_E_8_	−13.6 ± 0.5	123.3 ± 19.2	−3.5 ± 1.3	162.6 ± 22.0
EC-R_12–14_E_3_	−51.9 ± 2.2	19.9 ± 2.3	−28.7 ± 1.1	18.1 ± 1.3
EC-R_12–14_E_10_	−9.0 ± 1.1	5.6 ± 0.4	−2.4 ± 1.3	6.7 ± 0.1
AO-R_12_	−27.8 ± 1.5	52.4 ± 0.8	−11.0 ± 0.6	195.0 ± 4.5
AO-R_14_	−14.7 ± 1.3	8.8 ± 0.1	−6.9 ± 0.7	7.1 ± 1.1
AOP-_Cocoamido_	−12.7 ± 0.2	54.1 ± 8.7	−9.1 ± 0.4	326.9 ± 19.8
EC-R_8_E_8_ + EC-R_12–14_E_10_	−11.3 ± 1.1	7.0 ± 0.5	−5.9 ± 0.6	6.4 ± 0.5
EC-R_8_E_8_ + AO-_Cocoamido_	−12.8 ± 1.1	123.4 ± 12.0	−7.1 ± 0.2	36.0 ± 1.1
AO-R_12_ + AO-_Cocoamido_	−23.3 ± 3.4	48.2 ± 8.2	−10.4 ± 2.1	68.6 ± 2.7
AO-R_14_ + AO-_Cocoamido_	−12.0 ± 1.3	10.8 ± 2.6	−10.0 ± 1.5	8.9 ± 1.6

**Table 4:** Toxicity values (95% CI) of individual surfactants to bacteria *P. putida* (exposure time: 16 h and microalgae *P. tricornutum* (exposure time: 72 h).

Surfactant	*EC*_50_, *P. putida* mgL^−1^	*EC*_50_, *P. tricornutum* mgL^−1^
EC-R_8_E_8_	443.95 ± 10.24	93.05 ± 8.21
EC-R_12–14_E_3_	331.54 ± 15.77	13.14 ± 2.25
EC-R_12–14_E_10_	370.37 ± 15.02	53.14 ± 1.22
AO-R_12_	93.48 ± 4.76	29.72 ± 2.91
AO-R_14_	28.8 ± 2.48	4.27 ± 1.03
AOP-_Cocoamido_	311.48 ± 9.54	57.66 ± 5.48

**Table 5:** Toxicity values and MTU parameters of surfactants mixtures (1:1 *w*/*w*) to bacteria *P. Putida* (exposure time 16 h).

Surfactant A	Surfactant B	*EC*_50_, *P. putida* mgL^−1^ (95% CI)	*TU_A_*	*TU_B_*	*TU_mix_*	*TU_r_*	Type of Action
EC-R_8_E_8_	EC-R_12–14_E_10_	414.71 ± 18.33	0.47	0.56	1.03	1.83	Concentration addition
EC-R_8_E_8_	AO-_Cocoamido_	461.74 ± 22.15	0.52	0.74	1.26	1.70	Less than additive (antagonism)
AO-R_12_	AO_-Cocoamido_	210.98 ± 11.49	1.13	0.34	1.47	1.30	Less than additive (antagonism)
AO-R_14_	AO_-Cocoamido_	41.89 ± 3.87	0.73	0.07	0.79	1.09	Response addition

**Table 6:** Toxicity values and MTU parameters of surfactants mixtures (1:1 *w*/*w*) to marine microalgae *P. tricornutum* (exposure time: 72 h).

Surfactant A	Surfactant B	*EC*_50_, *P. tricornutum* mgL^−1^ (95% CI)	*TU_A_*	*TU_B_*	*TU_mix_*	*TU_r_*	Type of Action
EC-R_8_E_8_	EC-R_12–14_E_10_	55.3 ± 10.10	0.30	0.52	0.82	1.57	Concentration addition
EC-R_8_E_8_	AO-_Cocoamido_	69.91 ± 4.45	0.38	0.61	0.98	1.62	Concentration addition
AO-R_12_	AO_-Cocoamido_	65.24 ± 14.70	1.10	0.57	1.66	1.52	Less than additive (antagonism)
AO-R_14_	AO_-Cocoamido_	12.58 ± 4.18	1.47	0.11	1.58	1.07	Response addition

## Data Availability

The data presented in this study are available upon request from the corresponding author.

## References

[1] Modor Intelligence (2022). Surfactants Market, Growth, Trend, COVID-19 Impact and Forecast (2022–2027). https://www.Mordorintelligence.Com/Industry-Reports/Surfactants-Market.

[2] Jardak K., Drogui P., Daghrir R. (2016). Surfactants in Aquatic and Terrestrial Environment: Occurrence, Behavior, and Treatment Processes. Environ. Sci. Pollut. Res..

[3] Aloui F., Kchaou S., Sayadi S. (2009). Physicochemical Treatments of Anionic Surfactants Wastewater: Effect on Aerobic Biodegradability. J. Hazard. Mater..

[4] Nazari M., Kurdi M., Heerklotz H. (2012). Classifying Surfactants with Respect to Their Effect on Lipid Membrane Order. Biophys. J..

[5] Pereira L.C., de Souza A.O., Bernardes M.F.F., Pazin M., Tasso M.J., Pereira P.H., Dorta D.J. (2015). A Perspective on the Potential Risks of Emerging Contaminants to Human and Environmental Health. Environ. Sci. Pollut. Res..

[6] Badmus S.O., Amusa H.K., Oyehan T.A., Saleh T.A. (2021). Environmental Risks and Toxicity of Surfactants: Overview of Analysis, Assessment, and Remediation Techniques. Environ. Sci. Pollut. Res..

[7] Muherei M.A., Junin R. (2008). Mixing Effect of Anionic and Nonionic Surfactants on Micellization, Adsorption and Partitioning of Nonionic Surfactant. Mod. Appl. Sci..

[8] Salager J.-L., Marquez R., Bullon J., Forgiarini A. (2022). Formulation in Surfactant Systems: From-Winsor-to-HLDN. Encyclopedia.

[9] Liang L.X., Dong P., Zhou Y., Zhang L., Qian Z., Geiger S.D., Bingheim E., Tang X., Wu Y., Lv J. (2023). Joint Effects of Per- and Polyfluoroalkyl Substance Alternatives and Heavy Metals on Renal Health: A Community-Based Population Study in China. Environ. Res..

[10] Zhang F., Wang Z., Peijnenburg W.J.G.M., Vijver M.G. (2022). Review and Prospects on the Ecotoxicity of Mixtures of Nanoparticles and Hybrid Nanomaterials. Environ. Sci. Technol..

[11] Hu G., Wang H., Wan Y., Zhou L., Wang Q., Wang M. (2022). Combined Toxicities of Cadmium and Five Agrochemicals to the Larval Zebrafish (Danio Rerio). Sci. Rep..

[12] Eom H., Kim S., Oh S.E. (2023). Evaluation of Joint Toxicity of BTEX Mixtures Using Sulfur-Oxidizing Bacteria. J. Environ. Manag..

[13] Martin O., Scholze M., Ermler S., McPhie J., Bopp S.K., Kienzler A., Parissis N., Kortenkamp A. (2021). Ten Years of Research on Synergisms and Antagonisms in Chemical Mixtures: A Systematic Review and Quantitative Reappraisal of Mixture Studies. Environ. Int..

[14] Sigurnjak Bureš M., Cvetnić M., Miloloža M., Kučić Grgić D., Markić M., Kušić H., Bolanča T., Rogošić M., Ukić S. (2021). Modeling the Toxicity of Pollutants Mixtures for Risk Assessment: A Review. Environ. Chem. Lett..

[15] Kar S., Leszczynski J. (2019). Exploration of Computational Approaches to Predict the Toxicity of Chemical Mixtures. Toxics.

[16] Fernández-Serrano M., Jurado E., Fernández-Arteaga A., Ríos F., Lechuga M. (2014). Ecotoxicological Assessment of Mixtures of Ether Carboxylic Derivative and Amine-Oxide-Based Non-Ionic Surfactants on the Aquatic Environment. J. Surfactants Deterg..

[17] Ríos F., Fernández-Arteaga A., Lechuga M., Fernández-Serrano M., Dino Bidoia E., Nallin Montagnolli R. (2018). Ecotoxicological Characterization of Surfactants and Mixtures of Them. Toxicity and Biodegradation Testing.

[18] García M.T., Campos E., Ribosa I. (2007). Biodegradability and Ecotoxicity of Amine Oxide Based Surfactants. Chemosphere.

[19] Lechuga M., Fernández-Serrano M., Jurado E., Núñez-Olea J., Ríos F. (2016). Acute Toxicity of Anionic and Non-Ionic Surfactants to Aquatic Organisms. Ecotoxicol. Environ. Saf..

[20] (2012). European Commission Scientific Committee on Health and Environmental Risk; Scientific Committee on Emerging and Newly Identified Health Risks; Scientific Committee on Consumer Safety. Toxicity and Assessment of Chemical Mixtures..

[21] Altenburger R., Nendza M., Schüürmann G. (2003). Mixture Toxicity and Its Modeling by Quantitative Structure-Activity Relationships. Environ. Toxicol. Chem..

[22] Bragin G.E., Davis C.W., Kung M.H., Kelley B.A., Sutherland C.A., Lampi M.A. (2020). Biodegradation and Ecotoxicity of Branched Alcohol Ethoxylates: Application of the Target Lipid Model and Implications for Environmental Classification. J. Surfactants Deterg..

[23] Jurado E., Fernández-Serrano M., Lechuga M., Ríos F. (2012). Environmental Impact of Ether Carboxylic Derivative Surfactants. J. Surfactants Deterg..

[24] Clark D.P., Pazdernik N.J. (2016). Basics of Biotechnology. Biotechnology.

[25] Mozejko-Ciesielska J., Singh V. (2021). Pseudomonas Putida–Based Cell Factories. Microbial Cell Factories Engineering for Production of Biomolecules.

[26] (1995). Water Quality—Pseudomonas Putida Growth Inhibition Test (Pseudomonas Cell Multiplication Inhibition Test).

[27] Feng W., Swift S., Singhal N. (2013). Effects of Surfactants on Cell Surface Tension Parameters and Hydrophobicity of *Pseudomonas Putida* 852 and *Rhodococcus Erythropolis* 3586. Colloids Surf. B.

[28] Kaczerewska O., Martins R., Figueiredo J., Loureiro S., Tedim J. (2020). Environmental Behaviour and Ecotoxicity of Cationic Surfactants towards Marine Organisms. J. Hazard. Mater..

[29] de Carvalho R.C., Feijão E., Matos A.R., Cabrita M.T., Novais S.C., Lemos M.F.L., Caçador I., Marques J.C., Reis-Santos P., Fonseca V.F. (2020). Glyphosate-Based Herbicide Toxicophenomics in Marine Diatoms: Impacts on Primary Production and Physiological Fitness. Appl. Sci..

[30] Duarte B., Feijão E., de Carvalho R.C., Duarte I.A., Silva M., Matos A.R., Cabrita M.T., Novais S.C., Lemos M.F.L., Marques J.C. (2020). Effects of Propranolol on Growth, Lipids and Energy Metabolism and Oxidative Stress Response of *Phaeodactylum Tricornutum*. Biology.

[31] Feijão E., Cruz de Carvalho R., Duarte I.A., Matos A.R., Cabrita M.T., Novais S.C., Lemos M.F.L., Caçador I., Marques J.C., Reis-Santos P. (2020). Fluoxetine Arrests Growth of the Model Diatom *Phaeodactylum Tricornutum* by Increasing Oxidative Stress and Altering Energetic and Lipid Metabolism. Front. Microbiol..

[32] Cruz de Carvalho R., Feijão E., Matos A.R., Cabrita M.T., Utkin A.B., Novais S.C., Lemos M.F.L., Caçador I., Marques J.C., Reis-Santos P. (2022). Ecotoxicological Effects of the Anionic Surfactant Sodium Dodecyl Sulfate (SDS) in Two Marine Primary Producers: *Phaeodactylum Tricornutum* and Ulva Lactuca. Toxics.

[33] (2004). Surface Active Agents. Determination of Surface Tension.

[34] (2006). Water Quality. Marine Algal Growth Inhibition Test with Skeletonema Costatum and Phaeodactylum Tricornutum.

[35] Ríos F., Fernández-Arteaga A., Lechuga M., Fernández-Serrano M. (2017). Ecotoxicological Characterization of Polyoxyethylene Glycerol Ester Non-Ionic Surfactants and Their Mixtures with Anionic and Non-Ionic Surfactants Environ. Sci. Pollut. Res..

[36] Chen F., Wu L., Xiao X., Rong L., Li M., Zou X. (2020). Mixture Toxicity of Zinc Oxide Nanoparticle and Chemicals with Different Mode of Action upon *Vibrio Fischeri*. Environ. Sci. Eur..

[37] Broderius S.J., Kahl M.D., Hoglund M.D. (1995). Use of Joint Toxic Response to Define the Primary Mode of Toxic Action for Diverse Industrial Organic Chemicals. Environ. Toxicol. Chem..

[38] Hsu J.C. (1996). Multiple Comparisons: Theory and Methods.

[39] Jurado E., Fernández-Serrano M., Núñez-Olea J., Luzón G., Lechuga M. (2009). Acute Toxicity and Relationship between Metabolites and Ecotoxicity during the Biodegradation Process of Non-Ionic Surfactants: Fatty-Alcohol Ethoxylates, Nonylphenol Polyethoxylate and Alkylpolyglucosides. Water. Sci. Technol..

[40] Perinelli D.R., Cespi M., Casettari L., Vllasaliu D., Cangiotti M., Ottaviani M.F., Giorgioni G., Bonacucina G., Palmieri G.F. (2016). Correlation among Chemical Structure, Surface Properties and Cytotoxicity of N-Acyl Alanine and Serine Surfactants. Eur. J. Pharm. Biopharm..

[41] Perinelli D.R., Cespi M., Lorusso N., Palmieri G.F., Bonacucina G., Blasi P. (2020). Surfactant Self-Assembling and Critical Micelle Concentration: One Approach Fits All?. Langmuir.

[42] Ma X., Li M., Xu X., Sun C. (2022). Coupling Effects of Ionic Surfactants and Electrolytes on the Stability of Bulk Nanobubbles. Nanomaterials.

[43] Ríos F., Fernández-Arteaga A., Fernández-Serrano M., Jurado E., Lechuga M. (2018). Silica Micro- and Nanoparticles Reduce the Toxicity of Surfactant Solutions. J. Hazard. Mater..

[44] Jurado E., Fernández-Serrano M., Núñez Olea J., Lechuga M., Jiménez J.L., Ríos F. (2012). Acute Toxicity of Alkylpolyglucosides to *Vibrio Fischeri*, *Daphnia Magna* and Microalgae: A Comparative Study. Bull. Environ. Contam. Toxicol..

[45] Goodarzi F., Zendehboudi S. (2019). Effects of Salt and Surfactant on Interfacial Characteristics of Water/Oil Systems: Molecular Dynamic Simulations and Dissipative Particle Dynamics. Ind. Eng. Chem. Res..

[46] Qv X.Y., Jiang J.G. (2013). Toxicity Evaluation of Two Typical Surfactants to *Dunaliella Bardawil*, an Environmentally Tolerant Alga. Environ. Toxicol. Chem..

[47] Jurado E., Fernández-serrano M., Ríos F., Lechuga M., Chamy R., Rosenkranz F. (2013). Aerobic Biodegradation of Surfactants. Biodegradation-Life of Science.

[48] Jackson M., Eadsforth C., Schowanek D., Delfosse T., Riddle A., Budgen N. (2016). Comprehensive Review of Several Surfactants in Marine Environments: Fate and Ecotoxicity. Environ. Toxicol. Chem..

[49] Mustapha D.S., Bawa-Allah K.A. (2020). Differential Toxicities of Anionic and Nonionic Surfactants in Fish. Environ. Sci. Pollut. Res..

[50] Rosen M.J., Li F., Morrall S.W., Versteeg D.J. (2001). The Relationship between the Interfacial Properties of Surfactants and Their Toxicity to Aquatic Organisms. Environ. Sci. Technol..

[51] Rincón-Romero J.F., Ríos F., Reyes-Requena A., Luzón-González G., García-López A.I. (2023). Surface and Thermodynamics Properties of Commercial Fatty-Alcohol Ethoxylate Surfactants. J. Mol. Liq..

[52] Ríos F., Lechuga M., Fernández-Serrano M., Fernández-Arteaga A. (2017). Aerobic Biodegradation of Amphoteric Amine-Oxide-Based Surfactants: Effect of Molecular Structure, Initial Surfactant Concentration and pH. Chemosphere.

[53] Roberts D.W., Costello J.F. (2003). Mechanisms of Action for General and Polar Narcosis: A Difference in Dimension. QSAR Comb. Sci..

[54] Joshi V.Y., Kadam M.M., Sawant M.R. (2007). Comparison of QSAR and QSPR Based Aquatic Toxicity for Mixed Surfactants. J. Surfactants Deterg..

[55] Könnecker G., Regelmann J., Belanger S., Gamon K., Sedlak R. (2011). Environmental Properties and Aquatic Hazard Assessment of Anionic Surfactants: Physico-Chemical, Environmental Fate and Ecotoxicity Properties. Ecotoxicol. Environ. Saf..

[56] Martins N., Pereira J.L., Antunes F.E., Melro E., Duarte C.M.G., Dias L., Soares A.M.V.M., Lopes I. (2018). Role of Surfactant Headgroups on the Toxicity of SLEnS-LAS Mixed Micelles: A Case Study Using Microtox Test. Sci. Total Environ..

